# A Therapeutic Strategy for Chemotherapy-Resistant Gastric Cancer via Destabilization of Both β-Catenin and RAS

**DOI:** 10.3390/cancers11040496

**Published:** 2019-04-08

**Authors:** Won-Ji Ryu, Jae Eun Lee, Yong-Hee Cho, Gunho Lee, Mi-kyoung Seo, Sang-Kyu Lee, Jeong-Ha Hwang, Do Sik Min, Sung Hoon Noh, Soonmyung Paik, Sangwoo Kim, Jae-Ho Cheong, Kang-Yell Choi

**Affiliations:** 1 Translational Research Center for Protein Function Control, Yonsei University, Seoul 03722, Korea; wjryu711@naver.com (W.-J.R.); bioyonghee@naver.com (Y.-H.C.); sangklee@yonsei.ac.kr (S.-K.L.); jh_hwang19@naver.com (J.-H.H.); minds@pusan.ac.kr (D.S.M.); 2 Department of Biotechnology, College of Life Science and Biotechnology, Yonsei University, Seoul 03722, Korea; 3 Department of Surgery, Yonsei University College of Medicine, Seoul 03722, Korea; JELEE81@yuhs.ac (J.E.L.); SUNGHOONN@yuhs.ac (S.H.N.); 4 Department of Biomedical Systems Informatics, Yonsei University College of Medicine, Seoul 03722, Korea; GHLEE7@yuhs.ac (G.L.); MKSEO82@yuhs.ac (M.-k.S.); SWKIM@yuhs.ac (S.K.); 5 Graduate Program for Nanomedical Science, Yonsei University, Seoul 03722, Korea; 6 Brain Korea 21 PLUS Project for Medical Science, Yonsei University College of Medicine, Seoul 03722, Korea; 7 Department of Molecular Biology, College of Natural Science, Pusan National University, Pusan 46241, Korea; 8 Severance Biomedical Science Institute, Yonsei University College of Medicine, Seoul 03722, Korea; SOONMYUNGPAIK@yuhs.ac; 9 CK Biotechnology Inc., Rm 417, Engineering Research Park, 50 Yonsei Ro, Seodaemun-Gu, Seoul 03722, Korea

**Keywords:** gastric cancer, chemotherapy resistance, degradation of both β-catenin and RAS, gastric cancer patient-derived xenograft

## Abstract

Treatment of advanced gastric cancer patients with current standard chemotherapeutic agents frequently results in resistance, leading to poor overall survival. However, there has been no success in developing strategies to overcome it. We showed the expression levels of both β-catenin and *RAS* were significantly increased and correlated in tissues of 756 gastric cancer (GC) patients and tissues of primary- and acquired-resistance patient-derived xenograft tumors treated with 5-fluorouracil and oxaliplatin modulated with leucovorin (FOLFOX). On the basis of our previous studies, where small molecules to suppress colorectal cancer (CRC) via degrading both β-catenin and *RAS* were developed, we tested the effectiveness of KYA1797K, a representative compound functioning by binding axin, in the growth of GC cells. The efficacy test of the drugs using gastric tumor organoids of *Apc^1638N^* mice showed that the CD44 and ALDH1A3 cancer stem cell markers were induced by FOLFOX, but not by KYA1797K. KYA1797K also efficiently suppressed tumors generated by re-engrafting the FOLFOX-resistant patient-derived xenograft (PDX) tumors, which also showed resistance to paclitaxel. Overall, the small-molecule approach degrading both β-catenin and RAS has potential as a therapeutic strategy for treating GC patients resistant to current standard chemotherapies.

## 1. Introduction

Gastric cancer (GC) is one of the leading causes of cancer-related death globally, and *Helicobacter pylori* (*H. pylori*) infection, smoking, diet, and genetics are major causes of its occurrence [1,2,3,4]. Chemotherapies with or without radiation therapies accompanying surgery are the main treatment modalities for GC. However, many patients, particularly those at a late stage of the disease, experience recurrence after chemotherapy [5,6]. Although targeted therapies and immunotherapies are emerging as viable options for the treatment of such patients, resistance and recurrence are still major obstacles in the treatment of GC [7].

*H. pylori* infection, a major known risk factor for *GC* [8,9], and mutations of genes such as *adenomatosis polyposis coli* (*APC*), which are present in approximately 70% of patient with intestinal-type gastric adenoma [1,10], are known to activate the Wnt/β-catenin pathway, which is associated with the development and progression of GC. The aberrant activation of the RAS-ERK (extracellular signal regulated kinase) pathway by gene amplification and mutation of *RAS* and more frequently its upstream receptors such as ERBB2 and EGFR, frequently occurs in GC patients and plays roles in tumorigenesis [11,12]. Although *RAS* mutation is rare, its protein level was commonly found to be significantly increased via focal high-level amplification of its gene in tissues of GC patients [13,14,15]. Additionally, the GC patient with wild-type *KRAS*-amplified tumor had poorer survival compared to those without *KRAS* amplification [15]. Therefore, lowering the levels of both β-catenin and RAS could be a reasonable approach for controlling GC.

The Wnt/β-catenin and RAS-ERK pathways interact in colorectal cancer (CRC) tumorigenesis by gene mutations of the components in these two pathways such as the *APC* and *KRAS*, which critically enhanced the occurrence and progression of tumors [16,17,18]. The malignant phenotypes associated with both *APC* and *KRAS* mutations were attributed to increase in the levels of both β-catenin and mutant KRAS; these increases were caused by their stabilizations via *APC* loss, which inactivates GSK3β involved in phosphorylation-dependent β-catenin and Ras degradation [18,19]. In the present study, we found increases in the levels of both β-catenin and pan-RAS proteins, with a positive correlation between them, in tissues of 756 advanced GC (AGC) patients. Furthermore, their target genes are up-regulated in GC compared to normal gastric mucosa tissues. The roles of β-catenin and RAS in the resistance and recurrence of GC upon treatment with 5-fluorouracil and oxaliplatin modulated with leucovorin (FOLFOX), the 5-fluorouracil-based adjuvant chemotherapy, were indicated, given their associations with increases of both proteins in patient-derived xenograft (PDX) tumors established from tissues of AGC patient tumors. The CD44 and ALDH1A3 CSC markers were increased along with β-catenin and RAS in primary- and acquired-resistance PDX groups but not in the group that responded to FOLFOX treatment. 

We recently identified small molecules that degrade both β-catenin and Ras proteins by the screening of libraries of small molecules that inhibit the Wnt/β-catenin pathway [20,21]. KYA1797K, one of derivatives of initial hits, was characterized as a compound that interacts with the RGS domain of axin and effectively suppresses the growth of CRC by the activation of GSK3β via enhancement of the β-catenin destruction complex [20]. Therefore, in the present study, we tested the feasibility of using KYA1797K to suppress GC because of the increment of both β-catenin and pan-RAS proteins in tissues of AGC patients and PDX tumors. KYA1797K dose-dependently suppressed the transformation and growth of various GC cells with the decrease of both β-catenin and RAS. In addition, the resistance and recurrence upon treatment with chemotherapy were strongly associated with the enrichment of CD44 and ALDH1A3 CSC markers in the FOLFOX-treated tumor organoids (tumoroids) derived from *Apc*^1638N^ mouse gastric tumors. KYA1797K, but not FOLFOX, inhibited the enrichment of CSC markers with the critical inhibition of β-catenin and Ras, although both FOLFOX and KYA1797K reduced the size of the tumoroids. The effectiveness of KYA1797K on the suppression of gastric tumors, particularly those resistant to chemotherapy, was further confirmed by establishing PDX derived from acquired-resistant gastric tumor [22] against FOLFOX, followed by comparing its effectiveness with that of paclitaxel commonly used as next-line therapy for the recurrent AGC patients who had received first-line chemotherapy [23]. 

An approach of the suppression of CSCs via the degradation of both β-catenin and RAS by targeting the Wnt/β-catenin pathway has a potential as a therapeutic strategy to overcome the current limitations of resistance and recurrence after adjuvant chemotherapies in the clinical management of GC.

## 2. Results

### 2.1. Both β-Catenin and RAS Levels as well as CSC Markers were Increased in Tissues of AGC Patients

**Figure 1:**
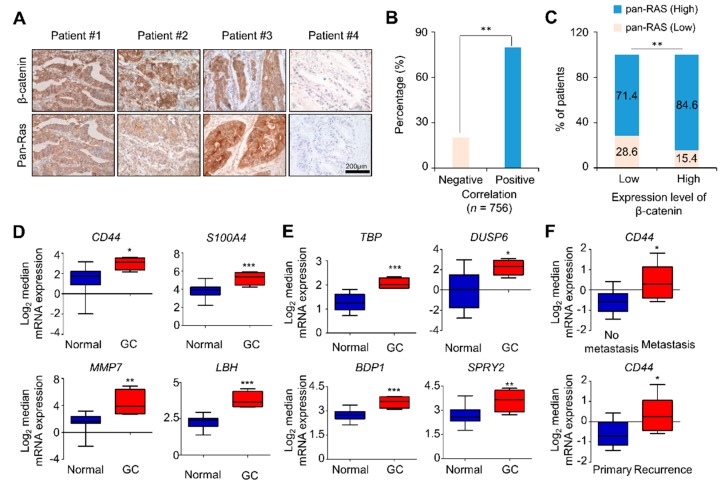
β-catenin and pan-RAS levels, and the expression of Wnt/β-catenin and RAS-ERK pathway target genes, including the CSC marker genes, were increased in tissues of GC patients. (**A**) Tumor tissues of 756 advanced gastric cancer (AGC) patients fixed on TMA slides were analyzed by 3,3′-diaminobenzidine staining using β-catenin or pan-RAS antibody. Representative images are presented. Scale bar represents 200 μM. (**B**) Correlations of the expression levels of pan-RAS and β-catenin in 756 AGC tissues are presented as percentages (*P* < 0.01). (**C**) Correlations of the expression levels of pan-RAS and β-catenin are presented on the basis of high and low patterns of these markers (*P* < 0.01, chi-squared test; comparing β-catenin low versus high). (**D**–**E**) Oncomine analysis of the DErrico database for the mRNA expression of Wnt/β-catenin (*CD44*, *S100A4*, *MMP7*, and *LBH*) and RAS-ERK (*TBP*, *DUSP6*, *BDP1*, and *SPRY2*) pathway target genes in GC tissues compared with normal gastric mucosa tissues (*n* = 69, DErrico Gastric statistics; *P* < 0.05 with Student’s *t*-test). (**F**) Oncomine analysis for the mRNA expression of CD44 in metastatic and recurring GC patient tissues compared with non-metastatic and primary tumor tissues, respectively (*n* = 43, Forster Gastric statistics; *P* < 0.05 with Student’s *t*-test).

To identify the involvement of the levels of β-catenin and RAS in the pathogenesis of GC, we analyzed tissue microarrays retaining tumor tissues of 756 AGC patients by immunohistochemical (IHC) analyses of β-catenin and pan-RAS. The correlations between β-catenin or pan-RAS expression and clinicopathological features of AGC are summarized in Supporting Information Table S1. The correlative activation status of β-catenin and RAS was analyzed by monitoring the subcellular localizations β-catenin and RAS (Figure S1) [24,25]. A strong positive correlation between the expression levels of β-catenin and pan-Ras was observed in approximately 79% of AGC patients. (Figure 1A,B; *P* < 0.05). The majority of AGC patient tumors (586 of 693 cases) was characterized by high expression levels of activated β-catenin and pan-Ras (Figure 1C; *P* < 0.05).

The clinical relevance of the activation of the two pathways in GC was further investigated by analyzing the expression patterns of target genes of the two pathways in tissues of GC patients from the database sets (DErrico and Forster Gastric statistics; https://www.oncomine.org). The expression of the Wnt/β-catenin pathway target genes, particularly those related to cancer, such as *CD44*, *S100A4*, *MMP7*, and *LBH*, was significantly increased in GC tumors compared with that in normal tissue (Figure 1D). The mRNA expressions of *TBP, DUSP6, BPD1,* and *SPRY2* genes, representative RAS target genes [26], were also elevated in the same database set (Figure 1E). Moreover, the CSC marker CD44, a target gene of Wnt/β-catenin pathway, was significantly induced in the tissues of GC patients, especially those who underwent metastasis and recurrence (Figure 1F).

To confirm the role of *APC* loss in the regulation of stability of RAS in the development of GC, we adopted *Apc*^1638N^ mouse as a model [27]. Our findings showed that both β-catenin and Ras levels were similarly increased, with an increment of Ki-67 in gastric tumors compared with adjacent normal tissue of *Apc*^1638N^ mice (Figure S2). In this animal model, increases of CD44 and ALDH1A3 CSC markers were also observed in gastric tumors compared with their levels in adjacent normal tissue (Figure S2). Overall, the expression of β-catenin and Ras as well as their downstream target genes, including several CSC markers of GC, was increased with a positive correlation in the tumor tissues of AGC.

### 2.2. The Wnt/β-catenin and RAS-ERK Pathways were Activated with Increases of both β-catenin and pan-RAS Levels in Tissues of Chemotherapy-Resistant PDX Tumor 

**Figure 2:**
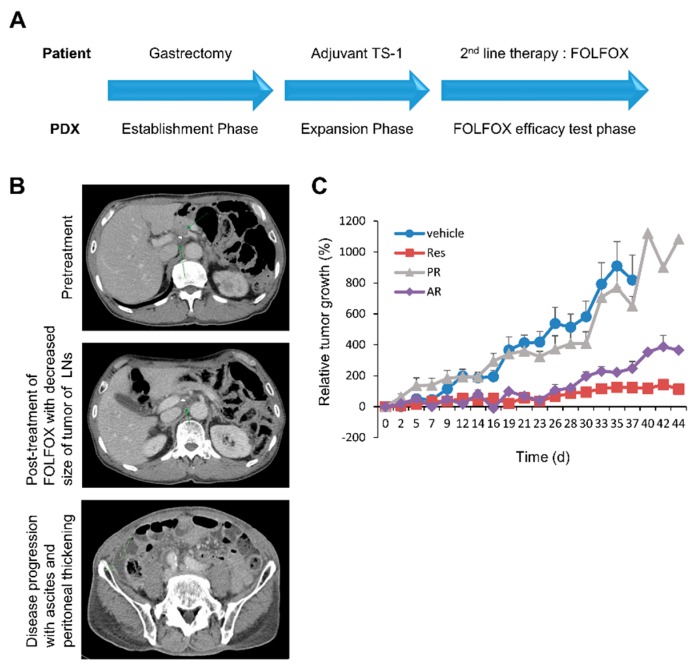
The poor clinical outcome of GC patient, and the heterogeneous response of patient-derived xenograft (PDX) tumors to 5-fluorouracil and oxaliplatin modulated with leucovorin (FOLFOX)**.** (**A**) Establishment and treatment of PDX tumors simultaneously with the clinical course of the patient. After gastrectomy, the primary tumor of the patient was engrafted into nude mice and allowed to expand for a FOLFOX-efficacy test. The patient in whom the adjuvant TS-1 chemotherapy has failed was administered into second-line therapy with FOLFOX accompanied by PDX. (**B**) CT images taken during the clinical course of the patient. The recurrent tumors (upper panel, arrow) showed a reduction in size after FOLFOX chemotherapy (middle panel, arrow). Subsequently, the peritoneal metastasis (lower panel, left arrow) grew, and ascites (lower panel, right arrow) developed. Tumor-bearing immunodeficient mice (*n* = 8 vehicle and *n* = 9 FOLFOX mice in each treatment group) were treated when the tumor had reached a volume of 100–200 mm^3^. FOLFOX (5-fluorouracil at 15 mg/kg and oxaliplatin at 5 mg/kg) was administered as described in the Methods section. (**C**) The reanalyzed tumor growth kinetics (*n* = 9 vehicle, *n* = 3 Res, *n* = 3 PR and *n* = 2 AR) from the FOLFOX-treatment experiment (Figure S3a).

To identify the involvement of the levels of β-catenin and pan-RAS in GC patients treated with chemotherapies, we exploited a PDX model treated with standard regimen chemotherapy. The PDX tumors were established from tumor tissues of a patient with primary gastric carcinoma after gastrectomy (Figure 2A). This patient was treated with adjuvant chemotherapy, but experienced tumor recurrence (Figure 2A,B; top). The patient was subsequently treated with FOLFOX and showed a substantial response to the therapy (Figure 2B; middle). Simultaneously, the PDX mice were treated with FOLFOX concurrently with the patient who provided the tumor; the tumor growth was markedly retarded in the FOLFOX-treated group, recapitulating the clinical response in the patient (Figure 2A,C, Figure S3a). However, the patient eventually developed peritoneal metastasis as a consequence of resistance to FOLFOX (Figure 2B; bottom). We revisited the PDX treatment results and reanalyzed tumor growth kinetics upon FOLFOX treatment. Despite the decreases in the overall growth and weight of PDX tumors due to FOLFOX treatment, the tumors of the FOLFOX-treated PDX group showed heterogeneous behaviors and were classified into three different groups: responder (Res), primary-resistance (PR), and acquired-resistance groups (AR) (Figure 2C).

**Figure 3:**
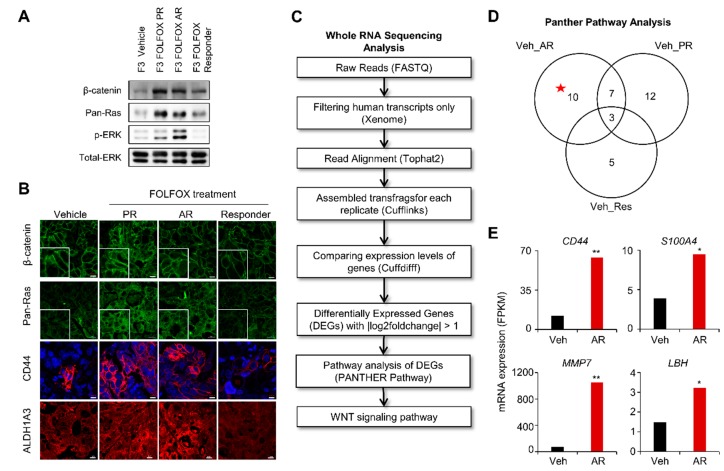
GC PDX tumors with acquired resistance to FOLFOX show increase of β-catenin and pan-Ras, and the activation of CSCs markers. (**A**) Immunoblot (IB) analyses using whole cell lysates (WCLs) of patient-derived xenograft (PDX) tumors treated with either vehicle or FOLFOX (*n* = 9 vehicle, *n* = 3 Res, *n* = 3 PR, and *n* = 2 AR). Immunoblotting was performed using the indicated antibodies as described in the Methods section. (**B**) Formalin-fixed 4-μm paraffin sections of the PDX tumors (**A**) were evaluated by immunohistochemical (IHC) analyses using anti-β-catenin, pan-Ras, CD44, or ALDH1A3 antibody. Nuclei were counterstained with4,6-diamidino-2-phenylindole (DAPI). Scale bar = 20 μm. Images were captured using confocal microscopy (LSM700; Carl Zeiss). (**C**) Flow chart of whole RNA sequence analysis. After FOLFOX treatment, the mRNA of PDX tumors was extracted and the change in the gene expression level was analyzed. (**D**) Venn diagram of exclusively enriched pathways in FOLFOX-treated groups compared with the vehicle group. Differential pathway enrichment was identified using statistical overrepresentation test of PANTHER (Protein ANalysis THrough Evolutionary Relationships) pathway analysis (Gene Expression Omnibus (GEO) accession number: GSE128967). (**E**) RNA-seq–based gene expression values (FPKM) for *CD44*, *S100A4*, *MMP7*, and *LBH* in vehicle and AR. Asterisks indicate statistically significant differences for gene expression in AR compared with vehicle. * *q* < 0.05, ** *q* < 0.005 versus control.

In the AR-PDX group, tumor growth was well suppressed up to 28 days after initial FOLFOX treatment; however, tumors started to re-grow despite continued treatment, mirroring the clinical course of the patient (Figure 2E). The levels of both β-catenin and RAS, as well as ERK activities were significantly increased in the PR- and AR-PDX tumors (Figure 3A). Interestingly, the level of ERK activity was much higher in the AR tumors than in the PR tumors, although the levels of β-catenin and pan-RAS were similarly increased in the PR tumors (Figure 3A). The increases of both β-catenin and pan-RAS in the PR and AR tumors were confirmed by IHC analyses (Figure 3B). In addition, CD44 and ALDH1A3 CSC markers were also increased in the PR and AR tumors (Figure 3B).

To gain insight into the mechanisms underlying the observed phenotype transitions, we analyzed and compared the transcriptomes between vehicle- and FOLFOX-treated groups. RNA sequencing was performed, followed by data analyses using PANTHER (Protein ANalysis THrough Evolutionary Relationships, http://pantherdb.org) 10.0 pathway annotation [28] (workflow outlined in Figure 3C). Through the statistical overrepresentation test in the PANTHER pathway analysis [29], the Wnt/β-catenin pathway was found to be an actionable therapeutic target of the 10 exclusively enriched signaling pathways with statistical significance in “vehicle versus AR” (Figure 3D, Figure S4). The role of Wnt/β-catenin signaling in the activation of CSCs and chemotherapy resistance was evidenced by increased *fragments per kilobase* of transcript per *million* mapped reads (FPKM) [30] values of *CD44, S100A4, MMP7,* and *LBH* genes in FOLFOX AR-PDX tumors (Figure 3E). 

### 2.3. Destabilization of both β-catenin and RAS Inhibited Transformation of GC Cells, and Suppressed Tumoroids Derived from Gastric Adenoma Tissues of Apc^1638N^ Mice

KYA1797K dose-dependently reduced levels of both β-catenin and RAS with the inactivation of ERK, downstream kinases of RAS, in GC cell lines (Figure S5a). Growth of these cells was also dose-dependently inhibited by treatment with KYA1797K (Figure S5b). Moreover, formation and growth rate of the colonies of GC cells were reduced by treatment with KYA1797K, as shown by reductions in both the number and size of colony (Figure S5c–e). Overall, reduction of both β-catenin and RAS by KYA1797K suppressed the growth and transformation of GC cells.

Treatment with KYA1797K also effectively suppressed the FOLFOX-induced increments of both β-catenin and RAS as well as the activities of ERK, respectively, in NCI-N87 cells (Figure S5f). Intriguingly, we did not observe differences in the changes of β-catenin and RAS levels and ERK activity upon co-treatment with FOLFOX and KYA1797K compared with those observed upon treatment with KYA1797K alone, which showed KYA1797K could override the effects of FOLFOX on β-catenin and RAS.

**Figure 4:**
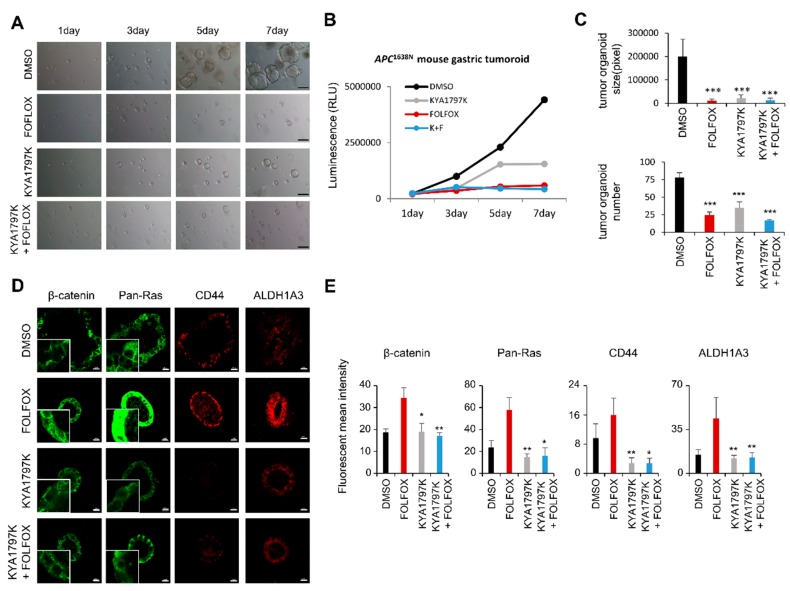
Effects of FOLFOX and KYA1797K on tumoroids from *Apc ^1638N^* mouse. Organoids derived from single *Apc ^1638N^* mouse tumor cells were dissociated and maintained every 7–10 days. (**A**) The effects of FOLFOX (5-fluorouracil at 10 μg/mL and oxaliplatin at 1 μg/mL) and KYA1797K (25 μM) on *APC^1638N^* mouse tumor-derived organoids (passage *n* = 6) were tested for 7 days. Representative images were captured every two days. Scale bar = 200 μm. (**B**) The number of viable cells was detected with reference to luminescence production (CellTiter-Glo® Luminescent Cell Viability Assay, Madison, Wisconsin, USA). (**C**) The sizes and numbers of *Apc^1638N^* mouse tumor-derived GC organoids in (**A**) were measured and quantified using the Image J program. Two-sided Student’s *t*-test was used to determine statistical significance. Error bars represent 95% confidence intervals (*n* = 3). (**D**) Immunocytochemical (ICC) analyses were performed by using β-catenin, pan-Ras, CD44, or ALDH1A3 antibody. Organoids were counterstained with DAPI. Boxes indicate the enlarged area, and dash line indicates the nucleus. Original photos with DAPI are shown in Supporting Information Figure S5. Images were captured using a Zeiss confocal microscope. Scale bar = 10 μm. (**E**) Mean fluorescence intensities of the markers were quantified for at least three different tumoroids using NIS-Elements AR 3.1 (Nikon; Melville, New York, USA). * *q* < 0.05, ** *q* < 0.005, *** *q* < 0.0005 versus control.

To validate the differential effects of FOLFOX and KYA1797K on the stemness of GC cells, we generated organoids using gastric adenoma tissues of *APC*^1638N^ mice, and tested the effects of these drugs. The growth of tumoroids was significantly suppressed by treatment with both FOLFOX and KYA1797K although no synergistic effects were observed when both of these drugs were administered together (Figure 4A,B). In addition, both number and size of tumoroids were decreased by treatment with KYA1797K or FOLFOX (Figure 4C). Again, the expression of both β-catenin and pan-Ras as well as CD44 and ALDH1A3 was significantly increased by FOLFOX treatment in the growth-suppressed tumoroids. However, none of these effects occurred in those treated with KYA1797K (Figure 4D,E).

### 2.4. KYA1797K Overcame Paclitaxel Resistance of PDX Tumors of FOLFOX-Resistant GC Tissues

Using a xenografted tumor with AR to FOLFOX, which exhibits higher activation of ERK, we tested whether KYA1797K can overcome this resistance by comparison with paclitaxel, the next-line therapy often prescribed to recurrent/metastatic GC patient. In this experimental design, we could test the feasibility of our approach destabilizing of both β-catenin and RAS on the GC resistant to the conventional therapy.

**Figure 5:**
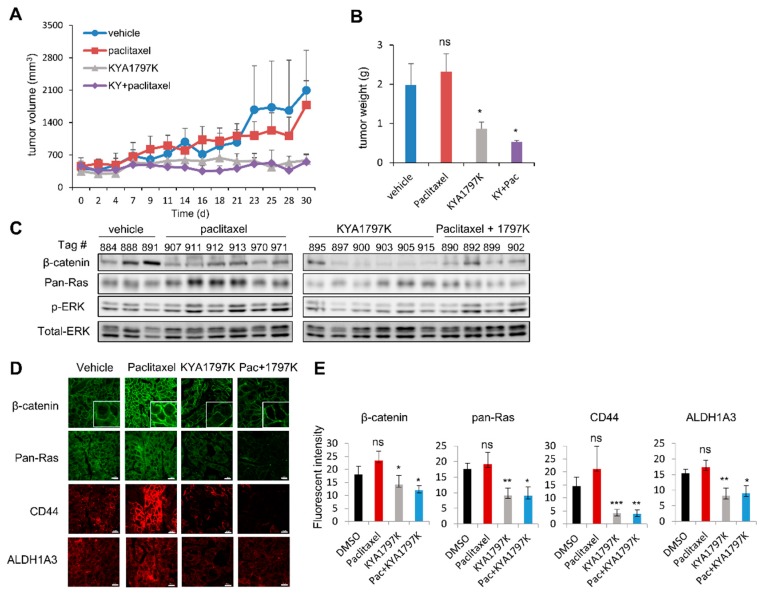
Effects of KYA1797K and paclitaxel on the growth of FOLFOX-resistant PDX tumors. Mice bearing FOLFOX-resistant tumors were treated with vehicle, or with paclitaxel (10 mg/kg), KYA1797K (25 mg/mL) or both, when the tumor reached a volume of 100–200 mm^3^. KYA1797K and paclitaxel were administered as described in the Method section. Subcutaneous tumor volumes were measured using calipers. (**A**) Tumor volumes of mice were measured for 30 days. (**B**) Tumor weight was measured at the time of sacrifice. (**C**) Immunoblot analyses using WCLs of PDX tumors (*n* = 3 vehicle, *n* = 6 paclitaxel, *n* = 8 KYA1797K, and *n* = 5 paclitaxel + KYA197K) (**A**), except for tumors that were too small (two tumors in the KYA1797K group and one tumor in the co-treatment group), to perform the above experiment with the indicated antibodies. (**D**,**E**) Formalin-fixed 4-μm paraffin sections of the PDX tumors (**A**) were evaluated by IHC analyses by using β-catenin, pan-Ras, CD44, or ALDH1A3 antibody and by mean fluorescence intensities of the markers for at least three different tissue samples using NIS-Elements AR 3.1 (Nikon; Melville, New York, USA). Two-sided Student’s *t*-test was used to determine statistical significance. Error bars represent 95% confidence intervals. * *P* < 0.05, ** *P* < 0.005., *** *P* < 0.0005. Scale bar = 10 μm.

The growth of xenografted tumors was not affected by treatment with paclitaxel, but was suppressed by treatment with KYA1797K alone or in combination with paclitaxel (Figure 5A). Both the weight and size of the tumors were significantly reduced with treatment of KYA179K; however, they were not significantly affected by treatment with paclitaxel (Figure 5B and Figure S7). Both β-catenin and pan-Ras were significantly reduced upon treatment with KYA1797K, although they mostly remained high or were even increased by treatment of paclitaxel (Figure 5C–E). Similar effects of KYA1797K on the specificities in the suppression of the expression of β-catenin and pan-RAS as well as CD44 and ALDH1A3 were confirmed by IHC analyses of the PDX tumor tissues (Figure 5C,E).

## 3. Discussion

Drug resistance and recurrence after initial chemotherapy are major causes of cancer patient mortality. However, no significant progress has been achieved, despite extensive efforts over the last few decades. In GC, the treatment of patients with the chemotherapeutic agents, such as 5-fluorouracil-based regimens, frequently results in the resistance and disease recurrence with a poor prognosis [31]. Therefore, the development of novel therapeutics to overcome conventional chemo-resistance is paramount for improving the clinical outcome of GC.

In the present study, we found that the levels of both β-catenin and RAS, the major components of the Wnt/β-catenin and Ras-ERK pathways, respectively, were up-regulated and positively correlated in tissues of AGC patient. The roles of these increased β-catenin and RAS levels in the resistance and reoccurrence of GC upon the chemotherapy were indicated by the up-regulations of both β-catenin and RAS in the PR- and AR-PDX tumors after FOLFOX treatment. The PR- and AR-PDX tumors also showed the activation of GC CSC markers such as CD44, which is a target gene of Wnt/β-catenin pathway as well as ALDH1A3, which is known to be upregulated via the mitogen-activated protein kinase (MEK)/ERK pathways [32,33,34]. The increase in ERK activity was more significant in the AR tumors than in the PR tumors, although both β-catenin and pan-RAS levels were similarly increased in the PR and AR tumors, indicating that the strong ERK activation may play a role in the acquired resistance to FOLFOX treatment. The role of FOLFOX in the CSC activation was confirmed by the transcriptional induction of *CD44* and *S100A4* in AR-PDX tumors compared with the levels in the vehicle. 

Considering the roles of β-catenin and RAS in the resistance and recurrence of GC after chemotherapy, we investigated the effectiveness of a compound that destabilize both β-catenin and RAS proteins via the activation of GSK3β through the specific binding of axin, a negative regulator of the Wnt/β-catenin pathway [20], on the suppression of GC, particularly in PDX model resistant against traditional chemotherapies related with the activation of CSCs.

A potential contribution of chemotherapy to the activation of CSCs was shown by the enrichment of CD44 and ALDH1A3 in the tumoroids derived from gastric tumor tissues from *Apc^1638N^* mice, which reduced their size upon FOLFOX treatment. The CSC induction by FOLFOX treatment indicates that the recurrence of tumors after initial response to FOLFOX treatment may be related to the activation of CSCs. Although both FOLFOX and KYA1797K similarly suppressed the growth of tumoroids, KYA1797K, which degrades β-catenin and RAS proteins, suppressed CSC activation of the tumoroids. Moreover, the induction of CSC markers by FOLFOX did not occur in the tumoroids co-treated with KYA1797K and FOLFOX.

The specific inhibition of both Wnt/β-catenin and Ras-ERK pathways on the suppression of CSCs involving the resistance and regrowth of GC was further confirmed using PDX tumors derived from a tumor with AR to FOLFOX. The second-line PDX tumors generated from first-line PDX tumors resistant to FOLFOX were not significantly suppressed by paclitaxel [35,36]. However, tumor growth was significantly reduced by KYA1797K treatment, inducing β-catenin and RAS destabilization. Here, the expression levels of CD44 and ALDH1A3 as well as β-catenin and pan-RAS were suppressed by KYA1797K but not by paclitaxel. Again, the suppression of tumor growth as well as the repression of all the biochemical markers did not show any difference upon co-treatment with paclitaxel, indicating that KYA1797K overrides the chemotherapy-induced stemness phenotype in resistant tumors.

In this study, we identified increased levels of both β-catenin and RAS in GC and revealed their roles in CSC activation involving the resistance to chemotherapy and recurrence after an initial response to this treatment. Furthermore, the effectiveness of a compound that destabilizes both β-catenin and RAS for suppressing of GCs, especially those resistant to chemotherapies due to CSCs, was proven using PDX models. Overall, the therapeutic approach degrading both β-catenin and RAS could be a potential approach for suppressing GCs, particularly those showing resistance and reoccurrence after initial treatment with standard chemotherapies.

## 4. Materials and Methods

### 4.1. Patients and TMA of AGC Patients

The present study included 756 GC patients who underwent surgical therapy at Yonsei University College of Medicine during the period from 2000–2003. Tissue samples of primary GC were obtained from these patients with written informed consent for it to be used for research purposes. Clinical data were retrieved from each patient’s medical records. Ethical approval to perform this study was obtained from the Institutional Review Board of Severance Hospital, Seoul, South Korea (4-2015-0616). This study did not include any sensitive information regarding patient privacy or identifiable information. Thus, a patient permission form was not required.

### 4.2. PDX Experiments

The detailed protocol for PDX establishment was as described previously [37]. Briefly, six-week-old male athymic nude mice (Japan SLC, Inc., Japan) or male NOG mice (NOD/Shi-scid, IL-2 Rγ null; CIEA, JAPAN) were used for each PDX model and were acclimatized for one week. The patient tumors were sliced into 3 × 3 × 3-mm^3^ fragments; then, these fragments were subcutaneously implanted into their flanks. The drug treatment was initiated when the mean tumor volume reached between 100–150 mm^3,^ and the mice were randomly assigned into specific treatment groups. The FOLFOX regimen was delivered with the following schedule: leucovorin (5 mg/kg/day) was injected intraperitoneally, followed by 5-FU (15 mg/kg/day) after 20 min once a day. Oxaliplatin (5 mg/kg/day) was injected intraperitoneally once a week. KYA1797K was injected intraperitoneally at a dose of 25 mg/mL/day daily, and paclitaxel was injected at 10 mg/mL once a week during 30 days. The sizes of the implanted tumors were measured three times a week using Vernier calipers, and the tumor volume was calculated as follows: (length × width^2^)/2. The mice were sacrificed, and the tumors were isolated, weighed, sliced, and fixed in formalin or liquid nitrogen for further analyses.

### 4.3. Whole RNA Sequencing Analysis of PDX Model

We used Xenome software tools to classify Xenograft RNA-seq human data and mouse (mm9) genome reference [38]. The classified FASTQ (https://en.wikipedia.org/wiki/FASTQ_format) data selected only the human genome. The paired-end reads were aligned to a human genome reference (UCSC hg19) using TopHat2 version 2.0.12 (https://ccb.jhu.edu/software/tophat/index.shtml) [39]. The aligned TopHat2 reads were assembled into transcripts using Cufflinks package 2.1.0 (http://cole-trapnell-lab.github.io/cufflinks/) [40], and Cuffdiff was used for gene expression value (fragments per kilobase of transcript per million mapped reads). Additionally, we used Cuffqant and Cuffnorm for the normalized expression levels from the set of RNA-Seq libraries. Using Cuffdiff, we analyzed the genes that were differentially expressed from the following comparisons: Vehicle versus Responder, Vehicle versus Primary Resistance, and Vehicle versus Acquired Resistance. Each group’s candidate genes were selected using the following thresholds: *q*-value < 0.05 and |log2 fold change (FC)| > 1. The statistical overrepresentation test module in PANTHER version 10.0 [41] was used to measure the significant ontology-based gene pathways. The pathways with a *p*-value < 0.05 were considered statistically significant. RNA-seq data for FOLFOX-treated PDX model (accession number: GSE128967) have been deposited in NCBI Gene Expression Omnibus database.

### 4.4. Cell Culture and Drug Treatment

Human GC cells (MKN74 and NCI-N87) were purchased from the Korea Cell Line Bank. MKN74, and NCI-N87 cells were cultured in RPMI (Gibco, Gaithersburg, MD) containing 10% fetal bovine serum (FBS; Gibco), 100 U/mL penicillin, and 100 µg/mL streptomycin (Gibco), and 5% CO_2_ at 37 °C. All chemicals were dissolved in dimethyl sulfoxide (DMSO; Sigma-Aldrich, St. Louis, MO, USA) for the in vitro studies, except for 5-FU (JW Pharma, Seoul, South Korea) and oxaliplatin (Sanofi-Aventis, St. Louis, MO, USA), which were dissolved in phosphate-buffered saline (PBS). Unless otherwise indicated, cells were treated with KYA1797K at a concentration of 25 µM for 24 h.

### 4.5. Tumor Organoid 

Isolated gastric tumors from *Apc^1638N^* mice were trypsinized for single-cell culture, and mixed with Matrigel (BD Biosciences, San Jose, CA), and plated in 48-well plates. After polymerization of Matrigel, gastric culture medium (Advanced DMEM/F12 supplemented with B-27 Supplement (50×), N2, 1 mM N-acetylcysteine (Invitrogen, 5823 Newton Dr, Carlsbad, CA), and gastrin (10 nM; Sigma-Aldrich) containing growth factors (50 ng/mL EGF, Peprotech, Rocky Hill, New Jersey; 1 mg/mL R-spondin1; 100 ng/mL Noggin, Peprotech; 100 ng/mL FGF10, Preprotech; and Wnt3A conditioned medium)) was overlain. For the first two days after seeding, the medium was also supplemented with 10 mM ROCK inhibitor Y-27632 (Sigma Aldrich) to avoid anoikis. Growth factors were added every second day, and the entire medium was changed every two days. For passage, gastric organoids were removed from the Matrigel, mechanically dissociated, and transferred to fresh Matrigel. Passage was performed every week with a 1:5–1:8 split ratio.

### 4.6. Mice and Maintenance 

C57BL/6J*Apc^1638N^* mice were obtained from Jackson Laboratory (Bar Harbor, Maine). Mice were housed in a micro-ventilation cage system with a computerized environmental control system (Threeshine Inc, Seoul, South Korea). Room temperature was maintained at 24 °C with a relative humidity of 40–70%. For mouse genotyping, the tail was used to extract genomic DNA; male mice were used for further animal experiments. For tissue preparation, stomach tissues from the mice were removed immediately after sacrificing the animals. The abdomens of the mice were cut open longitudinally and cleaned by flushing with PBS. After washing vigorously, resected tissues were fixed with 10% neutral formaldehyde (Sigma-Aldrich) for 24–48 h at 4 °C. Fixed tissues were embedded in paraffin according to standard procedures. The animal studies were approved and performed in accordance with the guidelines of the Institute of Health Guidelines for the Institutional Review Board of Severance Hospital, Yonsei University College of Medicine (Seoul, Korea).

### 4.7. Immunoblotting Analysis

Cells were washed in ice-cold PBS (Gibco, Gaithersburg, Maryland, USA) and lysed using radio-immunoprecipitation assay (RIPA) buffer containing 20 mM NaF, 1 mM sodium vanadate, 10 mM Tris-HCl at pH 7.5, 5 mM EDTA, 150 mM NaCl, 1% NP-40, 1 mM PMSF, and protease inhibitor cocktail (Millipore; Billerica, Massachusetts). The tissues were homogenized and dissolved in RIPA buffer, as described previously, and stored in a liquid nitrogen tank. Proteins were separated on a 6–12% sodium dodecyl sulfate-polyacrylamide gel (SDS-PAGE) and transferred to a nitrocellulose membrane (GE Healthcare Life Sciences, Pittsburgh, Pennsylvania). Immunoblotting was performed with the following primary antibodies: anti-pan-Ras monoclonal (clone Ras10; Millipore; MABS195; 1:3000), anti-β-catenin (Santa Cruz Biotechnology, Dallas, TX; sc-7199; 1:3000), anti-p-ERK (Cell Signaling Technology, Danvers, Massachusetts; #9101S; 1:1000), anti-ERK (Santa Cruz Biotechnology; sc-514302; 1:5000), anti-p-Akt (Cell Signaling Technology; #4060S; 1:1000), and anti-β-actin (Santa Cruz Biotechnology, sc-47778; 1:5000). Horseradish peroxidase-conjugated anti-mouse (Cell Signaling Technology; #7076; 1:3000) or anti-rabbit (Bio-Rad, Hercules, CA; 1:3000) secondary antibodies were used. Immunoblots were detected by enhanced chemiluminescence (Amersham Bioscience, Issaquah, WA) using a luminescent image analyzer (LAS-3000; Fuji Film, Tokyo, Japan).

### 4.8. Immunohistochemistry 

For IHC staining, 4-µm paraffin-embedded tissue sections were treated with citrate buffer (pH 6.0) and autoclaved for 15 min. The sections were then blocked with 5% bovine serum albumin and 1% normal goat serum (NGS; Vector Laboratories, Burlingame, CA) in PBS for 1 h for human and mouse tumor samples. For fluorescent IHC, sections were incubated with primary antibody (anti-β-catenin (BD Biosciences; #610154; 1:200), anti-pan-Ras monoclonal (clone Ras10; Millipore; MABS195; 1:100), anti-ALDH1A3 (Abcam, Cambridge, MA; ab129815; 1:100), or anti-CD44 (ProteinTech, Rosemont, IL; 1:100)) overnight at 4 °C, followed by incubation with anti-mouse Alexa Fluor 488 (Life Technologies, Camarillo, CA; A11008; 1:500) or anti-rabbit Alex Fluor 555 (Life Technologies; A21428; 1:500) secondary antibodies for 1 h at room temperature. The sections were counterstained with 4,6-diamidino-2-phenylindole (DAPI; Sigma-Aldrich) and mounted in Gel/Mount media (Biomeda Corporation, Foster City, CA). All processes were conducted in dark, humid chambers. A confocal microscope (LSM510; Carl Zeiss) was used to visualize the fluorescence signal at excitation wavelength of 488 nm (Alexa Fluor 488), 543 nm (Alexa Fluor 555), or 405 nm (DAPI). At least three fields per section were analyzed. Mean fluorescence intensities of the markers were measured at least three different tissue samples using NIS-Elements AR 3.1. (Laboratory Imaging, s.r.o., Praha, Czech Republic). For peroxidase IHC analysis, 3.45% H_2_O_2_ (Samchun Chemicals, Seoul, South Korea) was applied to sections to block endogenous peroxidase activity for 15 min. Before incubating sections with mouse primary antibody, mouse imunoglobuline G (IgG) was blocked using an M.O.M. Mouse IgG blocking kit (Vector Laboratories). Sections were incubated with anti-β-catenin, anti-pan-Ras, anti-ALDH1A3 or anti-CD44 primary antibody overnight at 4 °C, followed by incubation with biotinylated anti-mouse (Dako, Santa Clara, CA; E-0433; 1:300) or biotinylated anti-rabbit (Dako, E-0353; 1:300) secondary antibodies for 1 h at room temperature. The samples were then incubated in avidin biotin complex solutions (Vector Laboratories), stained with 3,3-diaminobenzidine (DAB; Dako) for maximum 5 min, and counterstained with Mayer’s hematoxylin (Muto, Bunkyou-ku, Tokyo, Japan). All incubations were performed in humid chambers. Signals were analyzed using a bright-field microscope (TE-2000U; Nikon). 

### 4.9. Cell Proliferation and Colony Formation Assays

To assay cell proliferation, MKN74 cells were plated at a density of 8 × 10^3^ cells/well, and NCI-N87 cells were seeded at a density of 1 × 10^4^ cells/well in a 24-well plate. The cells were then treated with 5 or 25 µM KYA1797K or with control (DMSO) for 72 h. Next, 3-(4, 5-dimethylthiazol-2-yl)-2-5-diphenyltetrazolium bromide (MTT; AMRESCO, Solon, OH) reagent was added to each well at a concentration of 0.25 mg/mL. After incubation for 1 h at 37 °C, insoluble purple formazan was obtained by incubating in 500 μL (24-well) or 200 μL (96-well) of DMSO for 20 min. The absorbance of the formazan product was determined at 590 nm every 24 h. For colony formation assays, cells were seeded in 12-well plates (500 cells/well for NCI-N87 and MKN74 cells). Control (DMSO) or KYA1797K was added to the cells with medium changes every three days until visible colonies formed. At the end of the experiment, cells were fixed in 4% paraformaldehyde (PFA) for 30 min and stained with 0.5% crystal violet in 20% ethanol for 30 min.

### 4.10. Statistical Analyses

Statistical analyses were performed using Microsoft Excel or GraphPad Prism 5 software (GraphPad Software, La Jolla, CA, USA). All data are represented as the mean ± standard deviation. For the TMA analysis of the AGC patients, statistical analysis was also performed using GraphPad Prism 5 software. The associations between β-catenin and pan-RAS staining were evaluated by the chi-squared test [42]. The statistical significance of differences was determined using Student’s *t*-tests, and statistically significant *p*-values are presented as follows: * *P* < 0.05; ** *P* < 0.005; and *** *P* < 0.0005.

## 5. Conclusions

Chemotherapy resistance and disease recurrence remains major causes of gastric cancer [43] patient mortality. We describe a possible relationship between Wnt/β-catenin and RAS/ERK pathways in GC patients and acquired-resistant GC PDX tumors against FOLFOX, 5-fluorouracil-based chemotherapy. RNA sequencing analysis also demonstrates that Wnt/β-catenin pathway is an actionable target pathway for overcoming the chemotherapy resistance. These provide that an approach targeting both Wnt/β-catenin and RAS/ERK pathways could be a novel therapeutic strategy in GC resistant to standard chemotherapy.

## Supplementary Materials

The following are available online at https://www.mdpi.com/2072-6694/11/4/496/s1. Figure S1: Correlative activation and increment of β-catenin and RAS protein level in GC tissue microarrays. Figure S2: The upregulated levels of β-catenin and Ras proteins as well as CSC markers in gastric tumors of *Apc*^1638N^ mice compared with those in adjacent normal tissue. Figure S3: The effect of FOLFOX on PDX models established from GC patient. Figure S4: Components of the Wnt/β-catenin pathway are enriched in AR-PDX tumors compared with vehicle tumors. Figure S5: Effects of KYA1797K on degradations of β-catenin and pan-Ras, proliferation and transformation of GC cells. Figure S6: Effects of FOLFOX and KYA1797K on the formation of tumoroids from *Apc ^1638N^* mice. Figure S7: The images of PDX tumors treated with vehicle, paclitaxel, KYA1797K, or co-treatment of paclitaxel and KYA1797K. Table S1. Patient characteristics and β-catenin or pan-Ras expression.
