# Supplementary material for: A Therapeutic Strategy for Chemotherapy-Resistant Gastric Cancer via Destabilization of Both β-Catenin and RAS
Source: Cancers (Basel). 2019 Apr 8;11(4):496. doi: 10.3390/cancers11040496 (PMC6521309; doi:10.3390/cancers11040496)
Supplement: Supplementary file 1 [file cancers-11-00496-s001.pdf]

Supplementary Materials

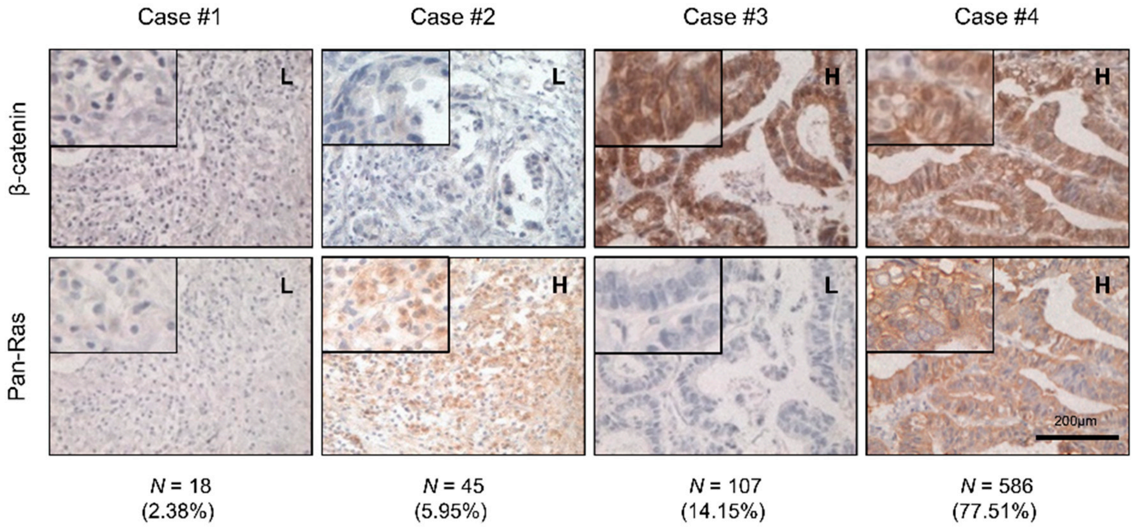

Figure S1. Correlative activation and increment of β-catenin and RAS protein level in GC tissue microarrays. Representative images showing the relationship between β-catenin and pan-RAS are presented. Low pattern = L, high pattern = H. Correlations of the activation of β-catenin and pan-RAS were analyzed by *chi*-squared test. The number and percentage of specimens in each category is given below each representative photomicrograph. Scale bar = 200 μm, and boxes show images at 400 × magnification.

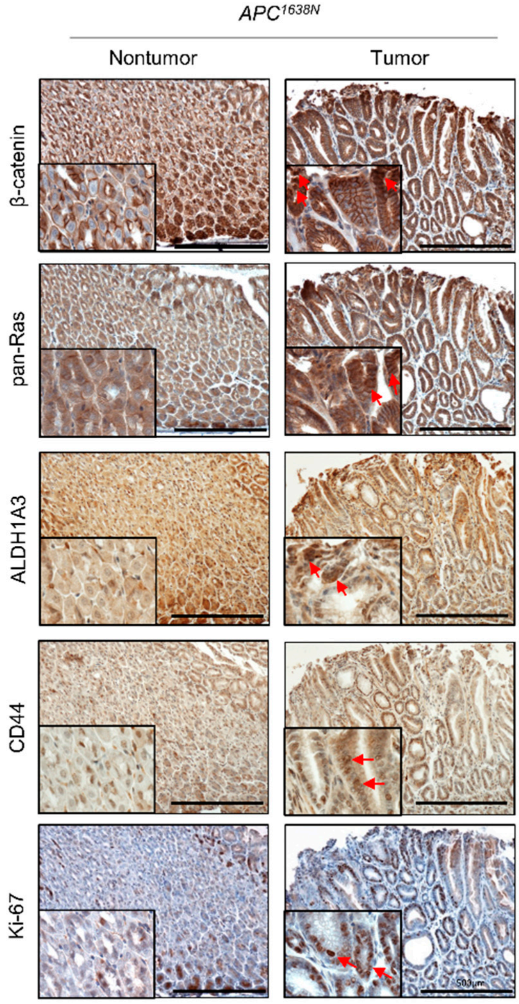

Figure S2. The upregulated levels of β-catenin and Ras proteins as well as CSC markers in gastric tumors of *Apc*[1638N] mice compared with those in adjacent normal tissue. Formaldehyde-fixed paraffin sections from gastric tissue of *Apc*[1638N] mice were subjected to IHC staining with the indicated antibodies specific for β-catenin, pan-Ras, CD44, ALDH1A3, or Ki67. Nuclei were counterstained with Mayer's hematoxylin. Scale bar = 500 μm.

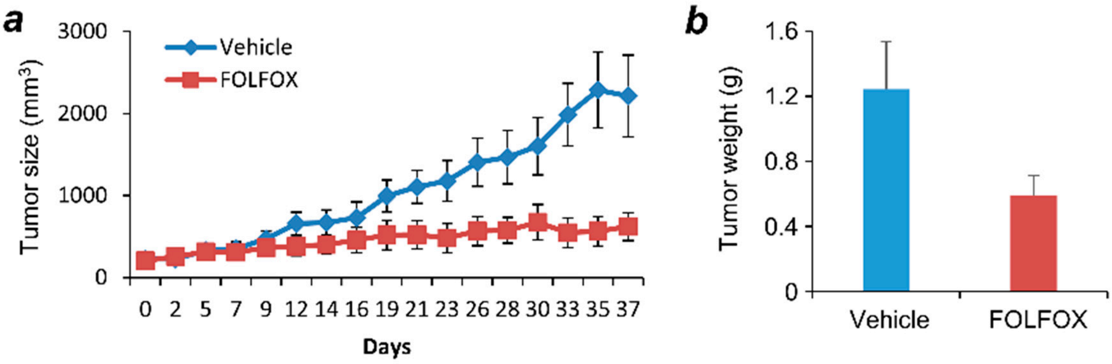

Figure S3. The effects of FOLFOX on the *in vivo* growth of GC patient's PDX. (**A**) Subcutaneous tumor volumes were measured by using calipers. The percentage change in tumor volume was calculated for each animal (n = 8 vehicle and n = 9 FOLFOX mice in each treatment group). Error bars represent means ± s.e.m. P-values were calculated by two-tailed t-test unless otherwise indicated. (**B**) The weights of the PDX tumors treated with vehicle or FOLFOX (P = 0.0539).

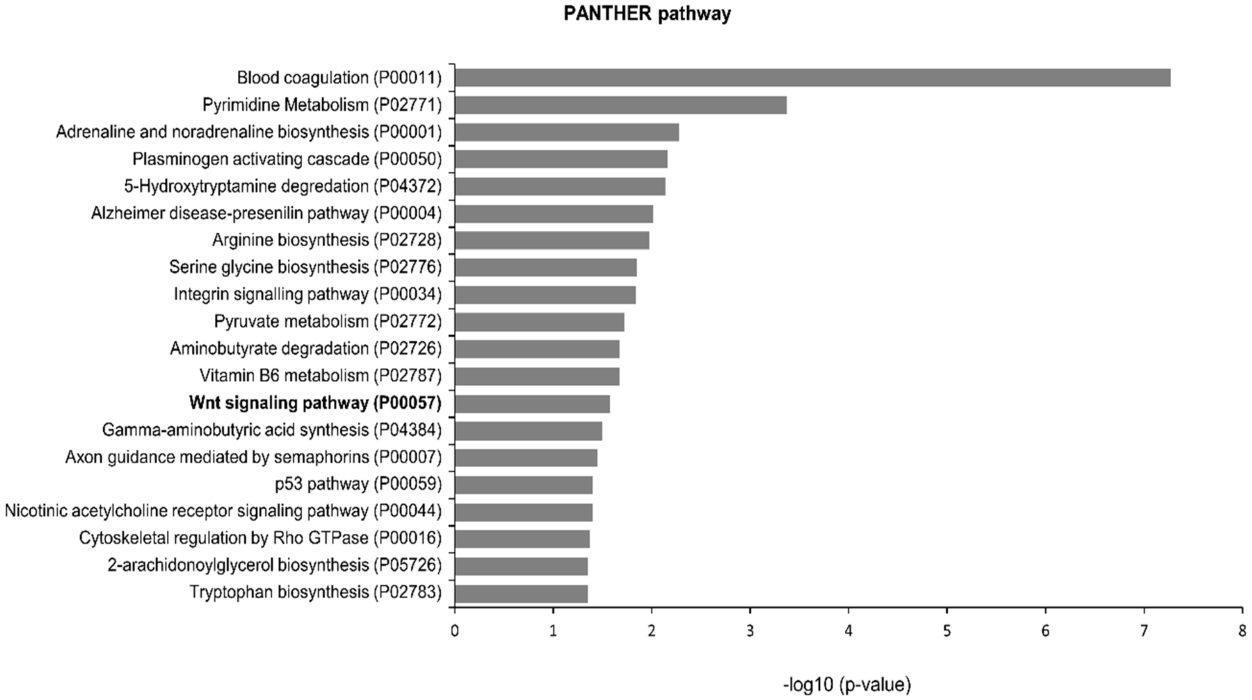

Figure S4. Components of the Wnt/β-catenin pathway are enriched in AR-PDX tumors compared with vehicle tumors. Pathway analysis using PANTHER databases. The significantly differentially expressed genes (DEGs) in "Veh vs. AR" described in Figure 3C, D were used for pathway analysis via PANTHER 10.0 pathway annotation. The most enriched pathways with statistical significance are shown. The x-axis shows the minus log 10 scale of *p*-values, whereas the y-axis shows each Gene Ontology term.

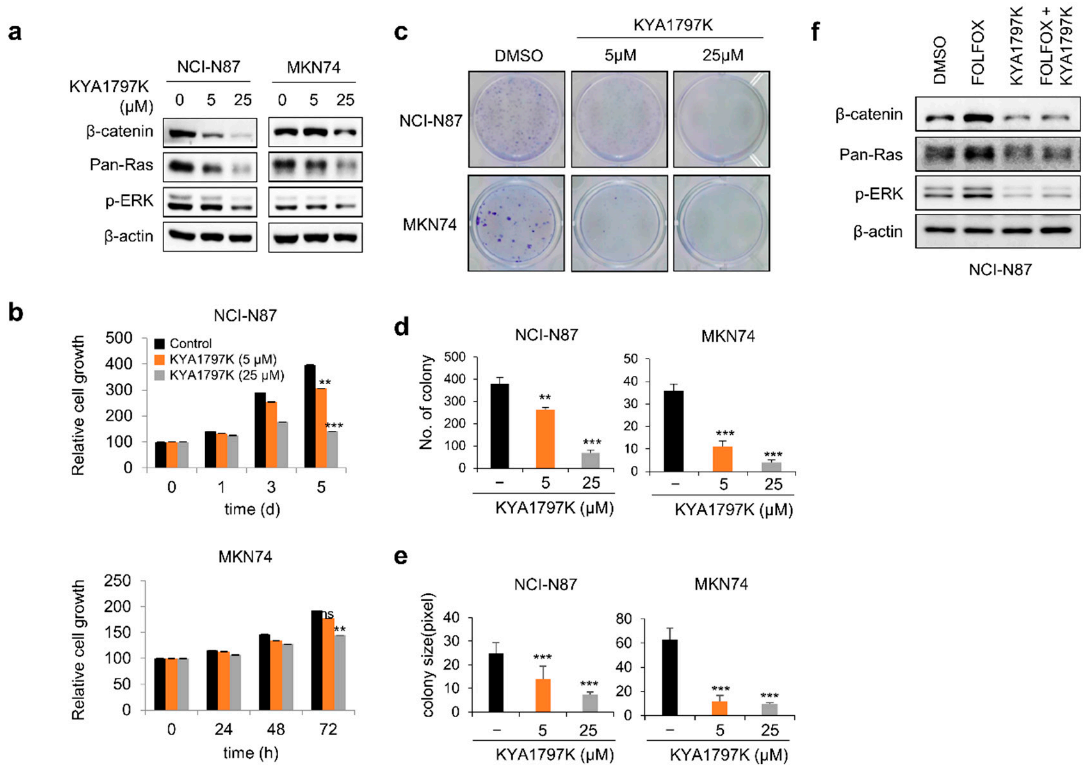

Figure S5. Effects of KYA1797K on degradations of β-catenin and pan-Ras, proliferation and transformation of GC cells. NCI-N87 or MKN74 GC cells were cultured as described in the Methods section. (**A**) The cells were cultured and treated with KYA1797K for 24 h. Whole-cell lysates (WCLs) were immunoblotted with the indicated antibodies. (**B**) NCI-N87 and MKN74 cells were treated with 5 μM or 25 μM KYA1797K. Cell proliferation was quantified using the MTT assay ($n = 3$). (**C–E**) The cells treated with KYA1797K were subjected to colony formation assay for 14 days. The foci in (**D**) and (**E**) were photographed and quantified from three independent experiments (mean ± s.d; $n = 3$). * $P < 0.05$, ** $P < 0.005$, *** $P < 0.0005$ versus control by Student's t-test. (**F**) IB analyses in NCI-N87 cells treated with vehicle, or with FOLFOX (5-FU at 10 μg/mL and oxaliplatin at 1 μg/mL), KYA1797K (25 μM) or both. IB analyses using WCLs were performed by using the indicated antibodies.

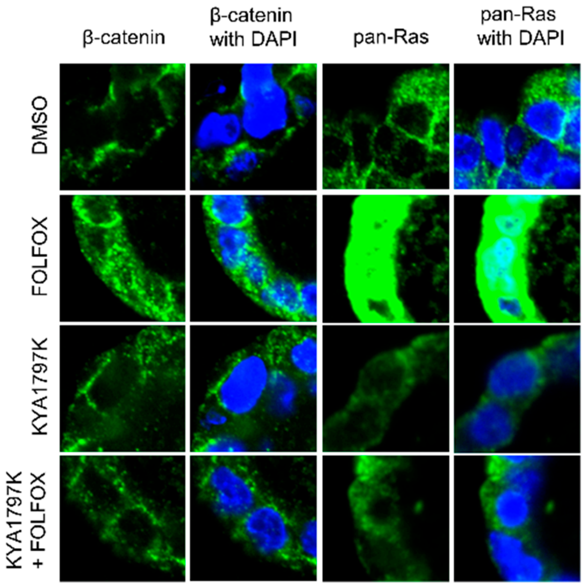

Figure S6. Effects of FOLFOX and KYA1797K on the formation of tumoroids from *Apc* ^{1638N} mice. Magnified images of β-catenin and pan-Ras in Figure 5D for clarifying the localizations of each markers. Nuclei were counter stained with DAPI.

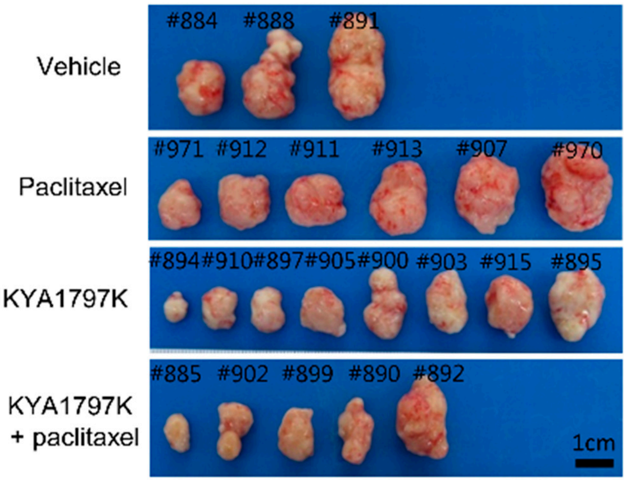

Figure S7. The images of PDX tumors treated with vehicle, paclitaxel, KYA1797K, or co-treatment of paclitaxel and KYA1797K. Tumor images were captured at the time of sacrifice. Scale bar = 1 cm.

Table S1. Patient characteristics and β-catenin or pan-Ras expression.

	**β-catenin**		*p-value*	**pan-Ras**		*p*-**value**
	**Low (*n* = 63)**	**High (*n* = 693)**		**Low (*n* = 125)**	**High (*n* = 631)**	
**Age (years)**			0.8606			0.1088
60≥	32 (50.8%)	360 (51.9%)		73 (58.4%)	319 (50.6%)	
60<	31 (49.2%)	333 (48.1%)		52 (41.6%)	312 (49.4%)	
**Sex**			0.0882			0.0464 *
Male	35 (55.6%)	459 (66.2%)		72 (57.6%)	422 (66.9%)	
Female	28 (44.4%)	234 (33.8%)		53 (42.4%)	209 (33.1%)	
**Lauren**			0.1212			<0.0001 *
Intestinal	27 (42.9%)	345 (49.9%)		41 (32.8%)	331 (52.5%)	
Diffuse	36 (57.1%)	322 (46.5%)		82 (65.6%)	276 (43.8%)	
Mix	0 (0%)	25 (3.6%)		2 (1.6%)	23 (3.7%)	
**pTstage**			0.8268			0.0013 *
pT1	0 (0.0%)	2 (0.3%)		2 (1.6%)	0 (0.0%)	
pT2	12 (19.0%)	106 (15.3%)		12 (9.6%)	106 (16.8%)	
pT3	24 (38.1%)	261 (37.7%)		56 (44.8%)	229 (36.3%)	
pT4	27 (42.9%)	324 (46.8%)		55 (44.0%)	296 (46.9%)	
**pNstage**			0.9749			0.2231
pN0	17 (27.0%)	180 (26.0%)		43 (34.4%)	154 (24.4%)	
pN1	13 (20.6%)	128 (18.5%)		20 (16.0%)	121 (19.2%)	
pN2	12 (19.0%)	145 (20.9%)		25 (20.0%)	132 (20.9%)	
pN3	12 (19.0%)	125 (18.0%)		20 (16.0%)	117 (18.5%)	
pN4	9 (14.3%)	115 (16.6%)		17 (13.6%)	107 (17.0%)	
**TNM stage**			0.4477			0.0257 *
Stage I	4 (6.3%)	62 (8.9%)		8 (6.4%)	58 (9.2%)	
Stage II	24 (38.1%)	214 (30.9%)		52 (41.6%)	186 (29.5%)	
Stage III	35 (55.6%)	417 (60.2%)		65 (52.0%)	387 (61.3%)	

Analysis by chi-square criterion or Fisher's exact test. * P < 0.05.
